# Using Wearable Sensors to Remotely Evaluate Progress on Goals Set by Patients With Dementia

**DOI:** 10.1093/geroni/igaa057.638

**Published:** 2020-12-16

**Authors:** Jennifer Freytag, He Zhou, Lilian Dindo, Aanand Naik

**Affiliations:** 1 Center for Innovations in Quality, Effectiveness, and Safety (iQuest), Houston, Texas, United States; 2 iCamp, Baylor College of Medicine, Houston, Texas, United States; 3 Michael E. DeBakey VA Medical Center, Houston, Texas, United States

## Abstract

Patient Priorities Care (PPC) is a communication framework designed to facilitate priorities-based conversations between clinicians and older adults with multiple chronic conditions. PPC focuses on collaboratively setting specific and measurable goals based on what matters most, and patients often set goals involving physical activity. Measuring goal achievement for patients with dementia is difficult because they often struggle to report activity accurately. In this pilot study, we assessed the feasibility of using a wearable sensor to evaluate patient-defined goal achievement. The wearable sensor measured daily mobility and sleep performance, including length of walking bouts, time sitting, postural transitions, and sleep duration. Patients wore pendant sensors for 48 hours at baseline (before PPC conversations) and 3-6 month follow-up. We present a case in which remote monitoring exhibited evidence of goal achievement and another case in which monitoring exhibited failure to achieve a goal. In the former, the patient set a goal of walking his dogs daily. At baseline, mobility performance suggested that he was not engaged in this activity. At follow-up, in contrast, all parameters showed accomplishment of the patient’s goal. In the latter case, the patient set a goal of maintaining his habit of walking a mile per day. At follow-up, sensor measures showed that the patient was not making progress on this goal. Conclusion: Remote monitoring using wearable sensors can provide objective information about goal achievement for patients with dementia. Sensor measurement can assist clinicians in evaluating goal achievement for patients with dementia and adjusting care based on these measures.

